# Supplementary material for: Pyruvate:ferredoxin oxidoreductase and low abundant ferredoxins support aerobic photomixotrophic growth in cyanobacteria
Source: eLife. 2022 Feb 9;11:e71339. doi: 10.7554/eLife.71339 (PMC8887894; doi:10.7554/eLife.71339)

Figure 1 - figure supplement 3 - source data – 1 (1) Original files of full, unedited gels

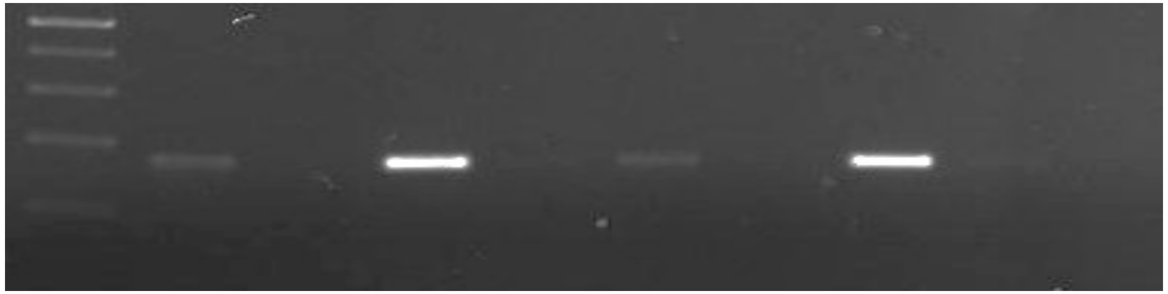

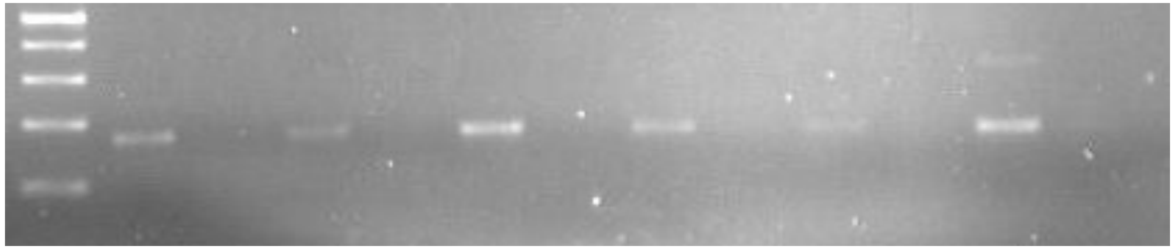

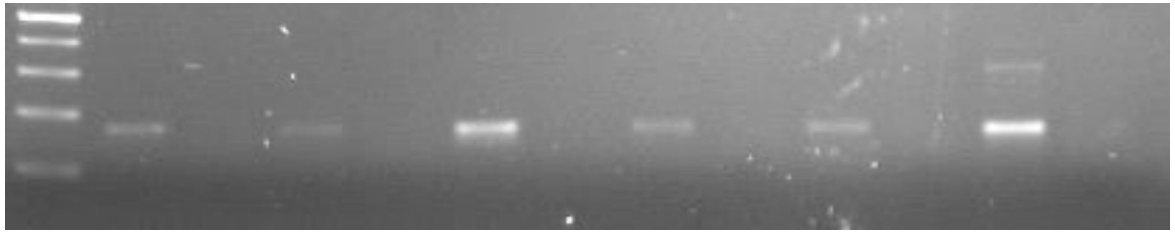

Figure 1 – figure supplement 3 - source data – 2 (2) Original files of full uncropped raw gel with relevant bands clearly labelled

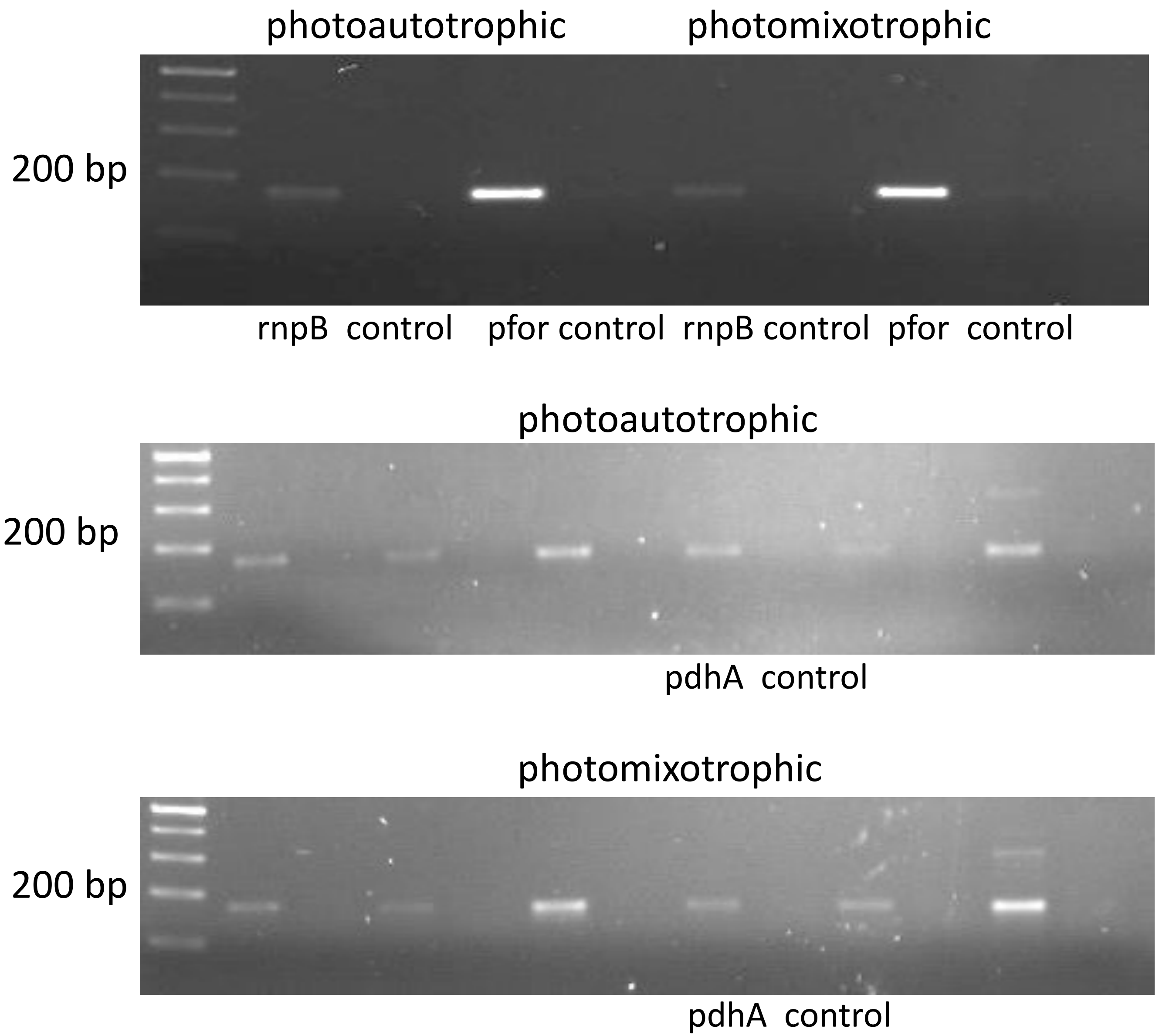

Supplement: Figure 1—figure supplement 3—source data 1. [file elife-71339-fig1-figsupp3-data1.zip › Figure_1_figure_supplement_3_source_data.pdf]
