# Supplementary material for: Pyruvate:ferredoxin oxidoreductase and low abundant ferredoxins support aerobic photomixotrophic growth in cyanobacteria
Source: eLife. 2022 Feb 9;11:e71339. doi: 10.7554/eLife.71339 (PMC8887894; doi:10.7554/eLife.71339)

Figure 3 - figure supplement 2 - source data - 1

(1) Original file of full uncropped raw, unedited gel

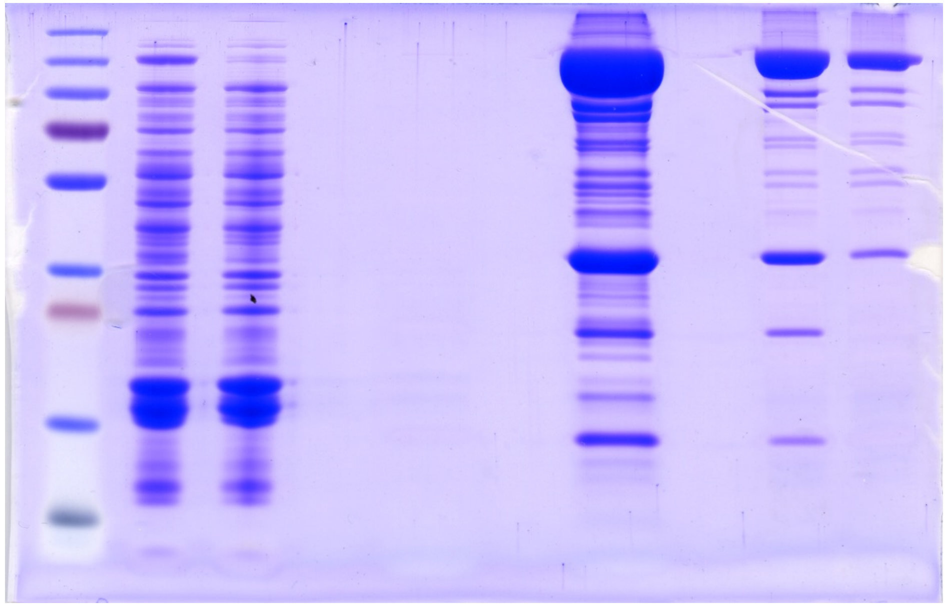

Figure 3 - figure supplement 2 - source data - 2

(2) Original file of full uncropped raw gel with relevant bands clearly labelled

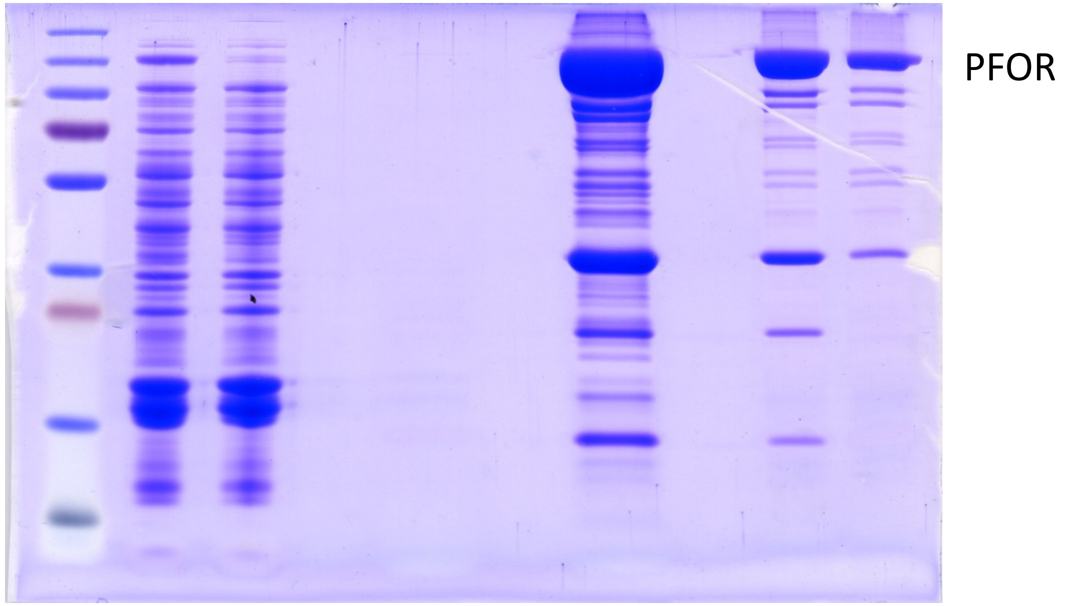

Supplement: Figure 2—figure supplement 2—source data 1. [file elife-71339-fig2-figsupp2-data1.pdf]
