# Supplementary material for: Pyruvate:ferredoxin oxidoreductase and low abundant ferredoxins support aerobic photomixotrophic growth in cyanobacteria
Source: eLife. 2022 Feb 9;11:e71339. doi: 10.7554/eLife.71339 (PMC8887894; doi:10.7554/eLife.71339)
Supplement: Figure 3—figure supplement 1—source data 1. [file elife-71339-fig3-figsupp1-data1.pdf]

Figure 3 - figure supplement 1A - source data – 1 and 2

(1) Original files of full uncropped raw, unedited gels

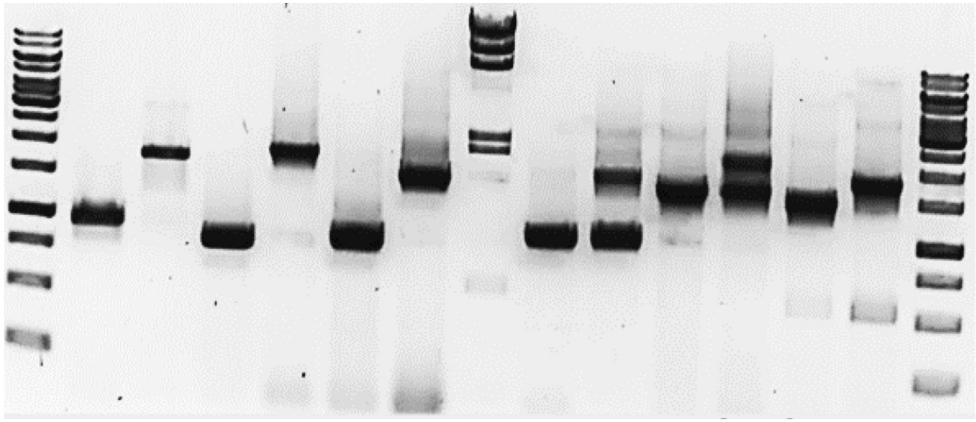

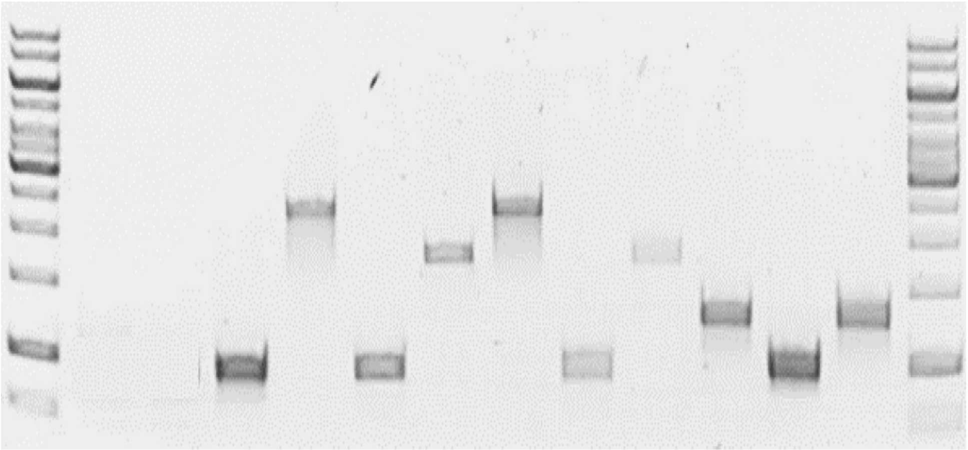

(2) Original files of full uncropped raw, unedited gels with relevant bands clearly labelled

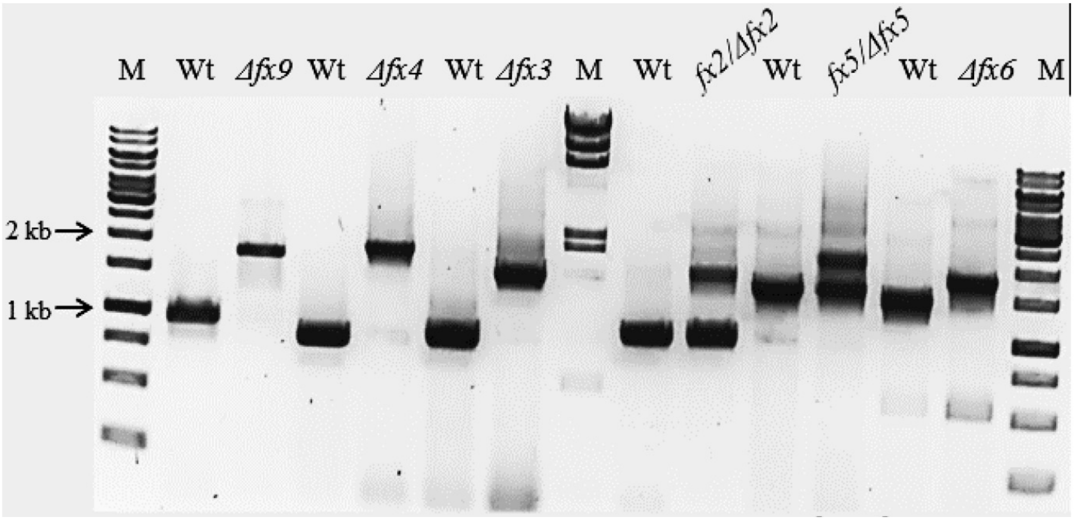

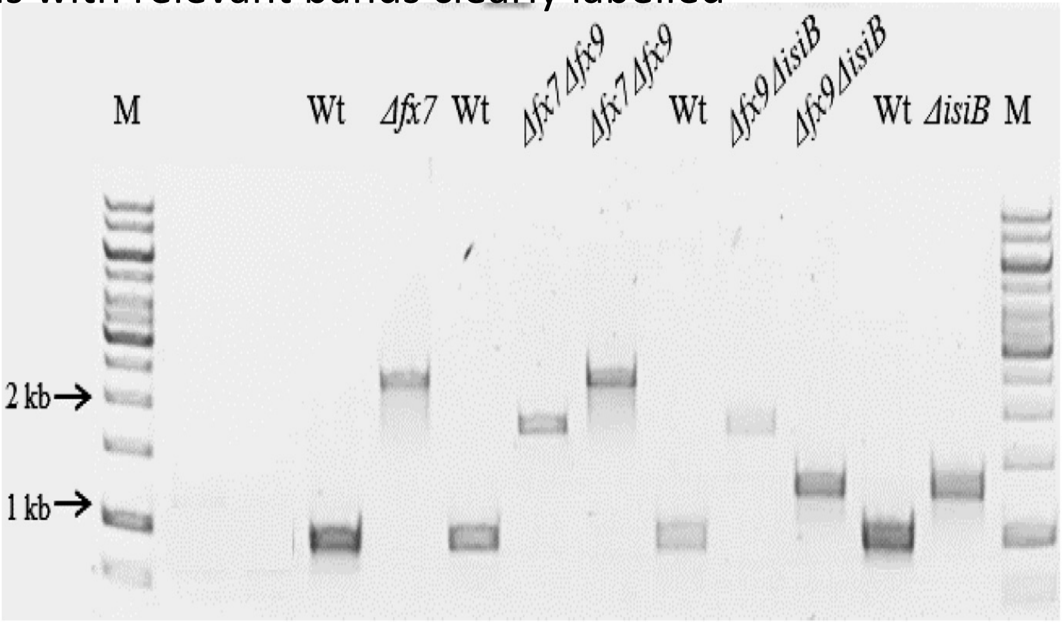

Figure 3 - figure supplement 1B - source data - 1

(1) Original files of full uncropped raw, unedited blots

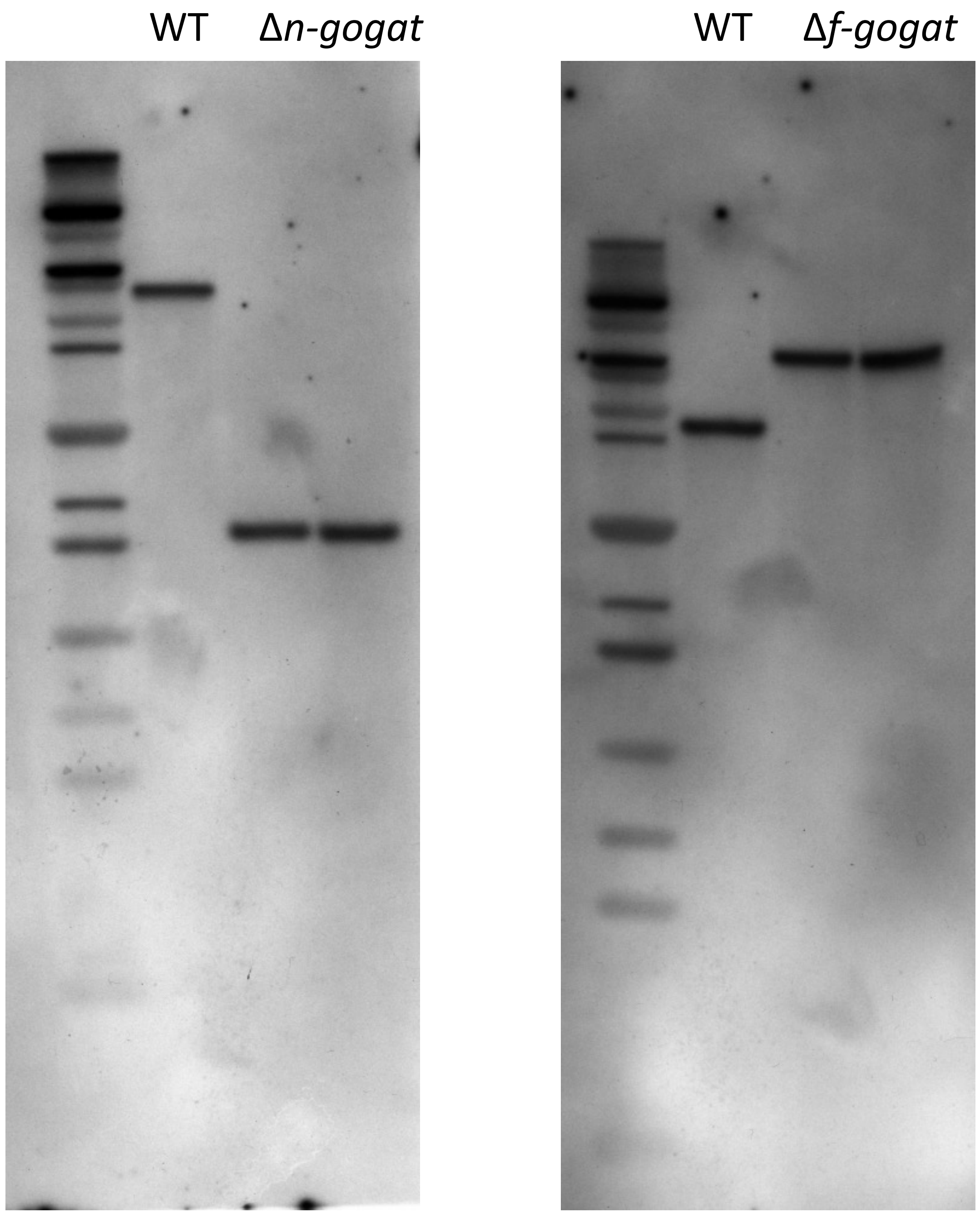

Southern blot of WT, Δ*n-gogat,* and Δ*f-gogat* deletion mutants. WT DNA and DNA from two different mutant clones were applied after HindIII digestion.

WT Δ*n-gogat*

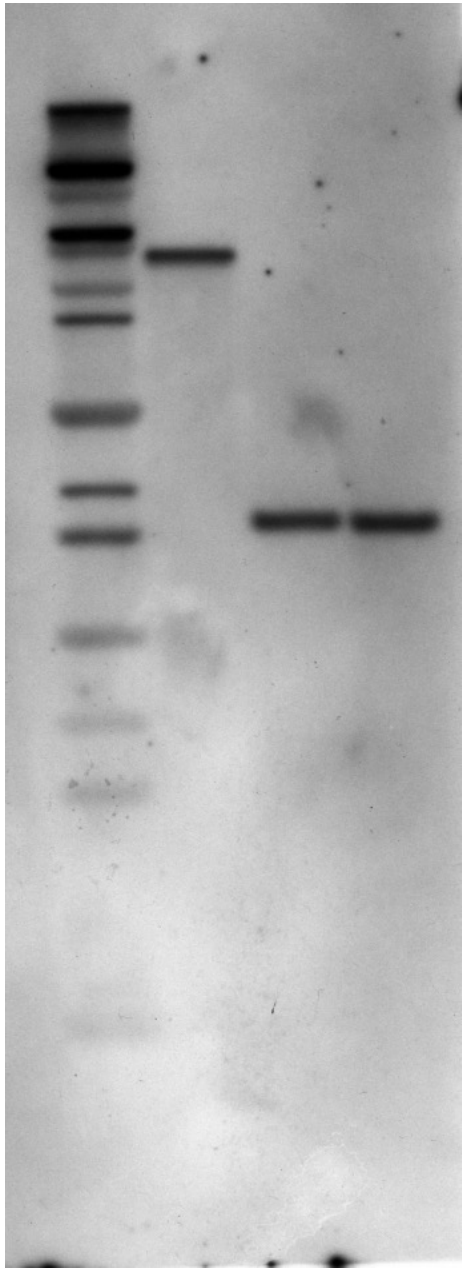

5857 bp

2073 bp

WT Δ*f-gogat*

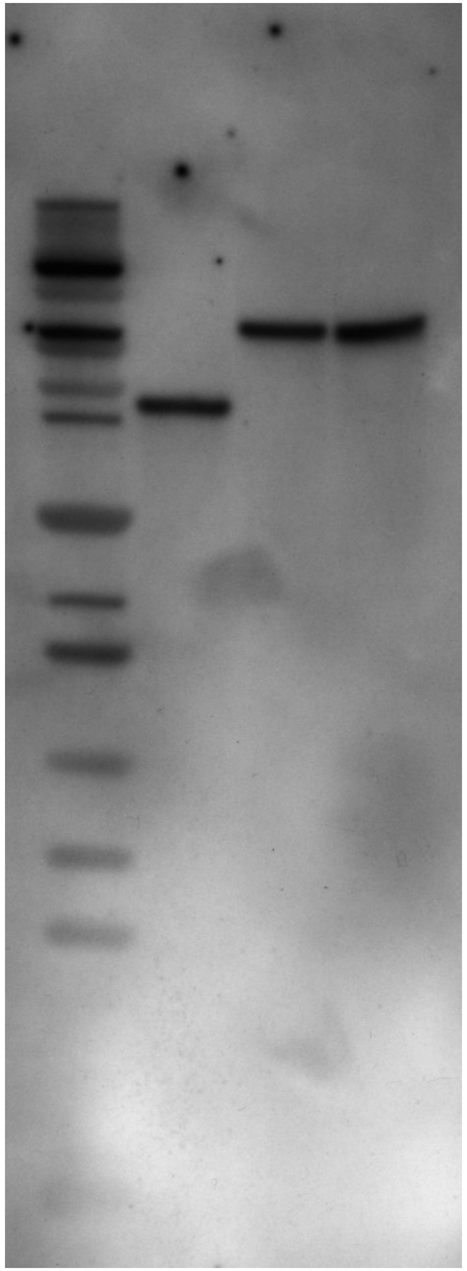

6506 bp

4882 bp

Figure 3 - figure supplement 1A - source data - 2

(2) Original files of full uncropped raw blots with relevant bands clearly labelled

Southern blot of WT, Δ*n-gogat,* and Δ*f-gogat* deletion mutants. WT DNA and DNA from two different mutant clones were applied after HindIII digestion. The sizes of the bands are indicated and correspond to those expected due to the mutation.
