# Supplementary material for: Pyruvate:ferredoxin oxidoreductase and low abundant ferredoxins support aerobic photomixotrophic growth in cyanobacteria
Source: eLife. 2022 Feb 9;11:e71339. doi: 10.7554/eLife.71339 (PMC8887894; doi:10.7554/eLife.71339)
Supplement: Supplementary file 1. — (a) List of primers used in this study to generate deletion strains and for RT-PCR. (b) List of Synechocystis strains and mutants used in this study. [file elife-71339-supp1.docx]

Supplementary Information

Pyruvate:ferredoxin oxidoreductase and low abundant ferredoxins support aerobic photomixotrophic growth in cyanobacteria

Yingying Wang^a^, Xi Chen^a^, Katharina Spengler^a^, Karoline Terberger^a^, Marko Boehm^a,b^, Jens Appel^a,b^, Thomas Barske^c^, Stefan Timm^c^, Natalia Battchikova^d^, Martin Hagemann^c^, Kirstin Gutekunst^a,b^

kirstin.gutekunst@uni-kassel.de

^a^Department of Biology, Botanical Institute, Christian-Albrechts-University, D-24118 Kiel, Germany; ^b^Department of Molecular Plant Physiology, Bioenergetics in Photoautotrophs, University of Kassel, D-34132 Kassel, Germany, ^c^Plant Physiology Department, University of Rostock, D-18059 Rostock, Germany, ^d^Department of Biochemistry, Molecular Plant Biology, University of Turku, FI-20014 Turku, Finnland

Supplementary File 1a: List of primers used in this study to generate deletion strains and for RT-PCR

Supplementary File 1b: List of *Synechocystis* strains and mutants used in this study

Supplementary File 1a: List of primers used in this study to generate deletion strains and for RT-PCR.

| Primer name | Sequence | Fragment amplified | Construct |
| --- | --- | --- | --- |
| pfor-1 | TGGGCTATCTCTTTCCCCGG | upstream recombination-site | Deletion of *pfor* (*sll0741*) |
| pforin1 | ATCTAATTTCTTTTTTCGTCGACAAGGGGTGATGGGATAAATGG |  |  |
| Em1 | GTCGACGAAAAAAGAAATTAGATAAA | Em-cassette |  |
| Em2 | GTCGACTTACTTATTAAATAATTTATAGC |  |  |
| pforin2 | AATTATTTAATAAGTAAGTCGACGGTCTATTCGGAAAATCGCTTT | downstream recombination-site |  |
| pfor-2 | ATTTTTGGTATTCATCTGAGTG |  |  |
| Fdx1.1 | CCGGTCCTTAAAACTCCCTT | upstream recombination site | Deletion of *fx1 (ssl0020)* |
| Fdx1in1 | TTGGCACCCAGCCTGCGCGAACAGTAGAGAGATTGCCTCAT |  |  |
| Sp-KG | TCGCGCAGGCTGGGTGCCAA | Sp-cassette |  |
| Sp-rev | GCCCTCGCTAGATTTTAATGCGGAT |  |  |
| Fdx1in2 | ATCCGCATTAAAATCTAGCGAGGGCGGTAATAATGCTGGCCATGG | downstream recombination site |  |
| Fdx1.2 | TTAATCTACCCTTCGTTTCCC |  |  |
| Fdx2.1 | CTCTCATATTCCGACCTACC | upstream recombination site | Deletion of *fx2 (sll1382)* |
| Fdx2in1 | ATCAGAGATTTTGAGACACAACGTGGTTATGGGCTGGTTTGAATCCA |  |  |
| Km1 | ccacgttgtgtctcaaaatctctgat | Km-cassette |  |
| Km2 | ATCGCCCCATCATCCAGCCAGAAAG |  |  |
| Fdx2in2 | CTTTCTGGCTGGATGATGGGGCGATGTAGGCTACAACTACAACCTG | downstream recombination site |  |
| Fdx2.2 | TCTGGGCAACGGCGTTTAAT |  |  |
| Fdx3.1 | CGTCTGCCGTACTGTTAGAT | upstream recombination site | Deletion of *fx3 (slr1828)* |
| Fdx3in1 | AGAGATTTATCTAATTTCTTTTTTCGTCGACCCATGGCAAAGCGGTAATAA |  |  |
| Em1 | GTCGACGAAAAAAGAAATTAGATAAA | Em-cassette |  |
| Em2 | GTCGACTTACTTATTAAATAATTTATAGC |  |  |
| Fdx3in2 | GCTATAAATTATTTAATAAGTAAGTCGACTTCGGCTGGAATTCTCCCTT | downstream recombination site |  |
| Fdx3.2 | GCAAAGACTCAAAGGACTGG |  |  |
| Fdx4.1 | CAATTACAGCCATCCTGTTTG | upstream recombination site | Deletion of *fx4 (slr0150)* |
| Fdx4in1 | TCAATAATATCGAATTCCTGCAGGAATGACCCAAACAATGGACT |  |  |
| Cm1 | CTGCAGGAATTCGATATTATTG | Cm-cassette |  |
| Cm2 | AAGCTTGATGGCGGCACCTCGCT |  |  |
| Fdx4in2 | AGCGAGGTGCCGCCATCAAGCTTAATGTTAGTCCAGCGGAGTT | downstream recombination site |  |
| Fdx4.2 | TTAGCAGGCAAGACCACACT |  |  |
| Fdx5.1 | CGATTCAGAACTCGGCATTG | upstream recombination site | Deletion of *fx5 (slr0148)* |
| Fdx5in1 | ATCAGAGATTTTGAGACACAACGTGGCATAATGGTGGCATGGTCATG |  |  |
| Km1 | ccacgttgtgtctcaaaatctctgat | Km-cassette |  |
| Km2 | ATCGCCCCATCATCCAGCCAGAAAG |  |  |
| Fdx5in2 | CTTTCTGGCTGGATGATGGGGCGATCGTTGACTCGTCTCACCATTG | downstream recombination site |  |
| Fdx5.2 | TCAGTGCTGGTAACACCATGG |  |  |
| Fdx6.1 | TTCTCCACGCAGTTGGTGAC | upstream recombination site | Deletion of *fx6* (*ssl2559*) |
| Fdx6in1 | GGTTCGTGCCTTCATCCGTCGACACCAGCATGGTATGGCGATC |  |  |
| Gm1 | GTCGACGGATGAAGGCACGAACC | Gm-cassette |  |
| Gm2 | GTCGACCGAATTGTTAGGTGGCG |  |  |
| Fdx6in2 | CGCCACCTAACAATTCGGTCGACTTGTCCGATGGAACCTAAGC | downstream recombination site |  |
| Fdx6.2 | AAGCTCTGGACGCCATTACC |  |  |
| Fdx7.1 | CCGTACTTAATGAATCGGCC | upstream recombination site | Deletion of *fx7 (Sll0662)* |
| Fdx7in1 | TTGGCACCCAGCCTGCGCGACAGGCACTCCAGCGTTGCAC |  |  |
| Sp-KG | TCGCGCAGGCTGGGTGCCAA | Sp-cassette |  |
| Sp-rev | GCCCTCGCTAGATTTTAATGCGGAT |  |  |
| Fdx7in2 | ATCCGCATTAAAATCTAGCGAGGGCTTAATTGGGTGATGGAATCT | downstream recombination site |  |
| Fdx7.2 | CTGAGTAGATTAATGTGGAC |  |  |
| Fdx8.1 | CGTTGGCTAGCATGTCACTG | upstream recombination site | Deletion of *fx8 (ssr3184)* |
| Fdx8in1 | TCAATAATATCGAATTCCTGCAGTAAGGGTAGCGGACGTTCAA |  |  |
| Cm1 | CTGCAGGAATTCGATATTATTG | Cm-cassette |  |
| Cm2 | AAGCTTGATGGCGGCACCTCGCT |  |  |
| Fdx8in2 | AGCGAGGTGCCGCCATCAAGCTTGGTTGGGAGGGGTCTAACTG | downstream recombination site |  |
| Fdx8.2 | CTCTGCCACTGTTAGGCTGC |  |  |
| Fdx9.1 | CGGAGGGGGAAACGGAAGAA | upstream recombination site | Deletion of *fx9 (slr2059)* |
| Fdx9in1 | ATCAGAGATTTTGAGACACAACGTGGGGCATTTGCACCGCACTACG |  |  |
| Km1 | ccacgttgtgtctcaaaatctctgat | Km-cassette |  |
| Km2 | ATCGCCCCATCATCCAGCCAGAAAG |  |  |
| Fdx9in2 | CTTTCTGGCTGGATGATGGGGCGATCATCTTTGCCGACTCCGCCA | downstream recombination site |  |
| Fdx9.2 | AATTCCAAAATAAATACCCC |  |  |
| isiB1 | ATGGATCATCCTCACACTTG | upstream recombination site | Deletion of flavodoxin (*isiB, sll0284*) |
| isiBin1 | GGTTCGTGCCTTCATCCGTCGACGATTACTGGAAAGTTACTAAGC |  |  |
| Gm1 | GTCGACGGATGAAGGCACGAACC | Gm-cassette |  |
| Gm2 | GTCGACCGAATTGTTAGGTGGCG |  |  |
| isiBin2 | CGCCACCTAACAATTCGGTCGACGCAATCCTAGGTAACCTAAG | downstream recombination site |  |
| isiB2 | CTGGTTTGTCATGGTAGGAG |  |  |
| pdhA1 | CAGGCGATCGCGTAACCGTTG | upstream recombination site | Deletion of *pdhA* (*slr1934*) |
| pdhAin1 | TTGGCACCCAGCCTGCGCGATCTATGCGAAGTCGGTCAGC |  |  |
| Sp-KG | TCGCGCAGGCTGGGTGCCAA | Sp-cassette |  |
| Sp-rev | GCCCTCGCTAGATTTTAATGCGGAT |  |  |
| pdhAin2 | ATCCGCATTAAAATCTAGCGAGGGCACGTTCACCGTTTGGGAGAA | downstream recombination site |  |
| pdhA2 | GACACCCAACCGCTAATGGA |  |  |
| NGOGATout1 | CTATAGGGCGAATTGGGTACCCAACTGAATTGCTTGGTGTTGT | upstream recombination site | Deletion of NADH-dependent GOGAT (*sll1502*) |
| NGOGATin1 | GGTTCGTGCCTTCATCCGTCGACGACCTTCGTGGCAGGGCAT |  |  |
| Gm1 | GTCGACGGATGAAGGCACGAACC | Gm-cassette |  |
| Gm2 | GTCGACGAATTGTTAGGTGGCG |  |  |
| NGOGATin2 | cgccacctaacaattcggtcgacGCGGCGTTTGAGGAGAAT | downstream recombination site |  |
| NGOGATout2 | AGGGAACAAAAGCTGGAGCT ATAGGTTGCAAACTTCATTAGCTA |  |  |
| FGOGATout1 | CTATAGGGCGAATTGGGTAC ACCATCAGGCTGGGCAATTTTGTT | upstream recombination site | Deletion of ferredoxin-dependent GOGAT (*sll1499*) |
| FGOGATin1 | TTGGCACCCAGCCTGCGCGA GTGGCAACAGAGGAGTTTGTCATA |  |  |
| Sp-KG | TCGCGCAGGCTGGGTGCCAA | Sp-cassette |  |
| Sp-rev | GCCCTCGCTAGATTTTAATGCGGAT |  |  |
| FGOGATin2 | ATCCGCATTAAAATCTAGCGAGGGC AGAAGACACTGACCTCTGTCTA | downstream recombination site |  |
| FGOGATout2 | AGGGAACAAAAGCTGGAGCT ACCGCAGGGACATTATGGGCTTA |  |  |
| pfor-tag1 | AGACCGTGTGCGAGCCAGCAAAGGGCCGATAGA | primer for RT-reaction | RT-PCR |
| pfor-tag2 | AGACCGTGTGCGAGCCAGCAA | primers for PCR |  |
| pfor-r | AACAATTTGGCCAGCTAACCGG |  |  |
| pdhA-tag1 | AGACCGTGTGCGACACGGGAATCCCTTCCCCAT | primer for RT-reaction |  |
| pdhA-tag2 | AGACCGTGTGCGACACGGGAAT | primers for PCR |  |
| pdhA-rev | TTACGTTTGCAGTACCTATCGA |  |  |
| rnpB-tag1 | AGACCGTGTGCGACACCAATCATGGGGCAGGAA | primer for RT-reaction |  |
| rnpB-tag2 | AGACCGTGTGCGACACCAATCA | primers for PCR |  |
| ndhD1out1 | CTATAGGGCGAATTGGGTACGACTATCTGGGTAGTATGAACACTT | upstream recombination site | pD1  Deletion of *ndhD1* (*slr0331*) |
| ndhD1in1 | ATCAGAGATTTTGAGACACAACGTGGGGTGGTGATAAAACCGGTGAGAA |  |  |
| Km1 | ccacgttgtgtctcaaaatctctgat | Km-cassette |  |
| Km2 | ATCGCCCCATCATCCAGCCAGAAAG |  |  |
| ndhD1in2 | CTTTCTGGCTGGATGATGGGGCGATGACCCCCATTTATCTACTCTCCAT | downstream recombination site |  |
| ndhD1out2 | AGGGAACAAAAGCTGGAGCTTTCTTGGTCGACTTAAAAACCAAT |  |  |
| ndhD2out1 | CTATAGGGCGAATTGGGTACCAGGCGGCATAGTCTTCGGAAAA | upstream recombination site | pD2  Deletion of *ndhD2* (*slr1291*) |
| ndhD2in1 | tcaataatatcgaaTTCCTGCAGAGTGTTCCAACATGGTAATAAGAA |  |  |
| Cm1 | CTGCAGGAATTCGATATTATTG | Cm-cassette |  |
| Cm2 | AAGCTTGATGGCGGCACCTCGCT |  |  |
| ndhD2in2 | AGCGAGGTGCCGCCATCAAGCTTTCAAAGTTCAACCCTAGTGATCTA | downstream recombination site |  |
| ndhD2out2 | AGGGAACAAAAGCTGGAGCTAACCGATGCCCACACCGGTCTGATT |  |  |

Supplementary File 1b: Liste of *Synechocystis* strains and mutants used in this study.

| Strain | Marker of genotype | *Synechocystis* WT background | Reference |
| --- | --- | --- | --- |
| WT |  | non-motile GT strain | Trautmann et al. 2012 |
| fx2/Δfx2 | sll1382::km^R^ | non-motile GT strain | Gutekunst et al. 2014 |
| Δfx3* | slr1828::em^R^ | non-motile GT strain | Gutekunst et al. 2014 |
| Δfx4* | slr0150::cm^R^ | non-motile GT strain | Gutekunst et al. 2014  *please note that the names of fx3 and fx4 are exchanged in Gutekunst et al. 2014 |
| fx5Δfx5 | slr0148::km^R^ | non-motile GT strain | This study |
| Δfx6 | ssl2559::gm^R^ | non-motile GT strain | This study |
| Δfx7 | sll0662::spec^R^ | non-motile GT strain | This study |
| Δfx9 | slr2059::km^R^ | non-motile GT strain | This study |
| ΔisiB | sll0284::gm^R^ | non-motile GT strain | Gutekunst et al. 2014 |
| Δfx7Δfx9 | sll0662::spec^R^, slr2059::km^R^ | non-motile GT strain | This study |
| Δfx7Δfx8Δfx9 | sll0662::spec^R^, ssr3184::cm^R^,  slr2059::km^R^ | non-motile GT strain | This study |
| Δfx9ΔisiB | slr2059::km^R^, sll0284::gm^R^ | non-motile GT strain | This study |
| Δn-gogat | *sll1502::gm^R^* | non-motile GT strain | This study |
| Δf-gogat | *sll1499::spec^R^* | non-motile GT strain | This study |
| ΔndhD1ΔndhD2 | slr0331::km^R^, slr1291::cm^R^ | non-motile GT strain | This study |
| Δhk | sll0593::spec^R^ | non-motile GT strain | Theune et al. 2021 |
| ΔglgP1ΔglgP2 | sll1356::km^R^, slr1367::spec^R^ | non-motile GT strain | Makowka et al. 2020 |
